# Microbial Inoculation during the Short-Term Composting Process Enhances the Nutritional and Functional Properties of Oyster Mushrooms (*Pleurotus ostreatus*)

**DOI:** 10.3390/life14020201

**Published:** 2024-01-31

**Authors:** Qiuying Wang, Minrui Zhao, Yiyang Wang, Zhenfei Xie, Shunyin Zhao, Shuning You, Qingjun Chen, Weiwei Zhang, Yong Qin, Guoqing Zhang

**Affiliations:** 1 College of Horticulture, Xinjiang Agricultural University, Urumqi 830052, China; 202140240004@bua.edu.cn (Q.W.); 202040240003@bua.edu.cn (Y.W.); 2 Beijing Key Laboratory for Agricultural Application and New Technique, College of Plant Science and Technology, Beijing University of Agriculture, Beijing 102206, China; 202130212047@bua.edu.cn (M.Z.); 202230212051@bua.edu.cn (Z.X.); 202230212071@bua.edu.cn (S.Z.); 202330212044@bua.edu.cn (S.Y.); 20036304@bua.edu.cn (Q.C.); 20238902@bua.edu.cn (W.Z.)

**Keywords:** *Pleurotus ostreatus*, composting cultivation, *Streptomyces thermoviolaceus*, nutritional composition, flavor compounds, antioxidant activity

## Abstract

In recent years, short-term composting techniques have been widely applied in oyster mushroom cultivation, but there is still a lack of systematic research on their impact on the nutritional and functional properties of fruiting bodies. In this study, the microbial inoculant *Streptomyces thermoviolaceus* BUA-FM01 (ST) was applied in the short-term composting process for oyster mushroom cultivation. The agronomic traits, nutritional composition, flavor compounds, and antioxidant activity of fruiting bodies from the first three flushes were evaluated. The results show that microbial inoculation significantly (*p* < 0.05) reduced the total carbon content and C/N ratio of the composted substrates and, furthermore, increased the total yield of the fruiting bodies. Moreover, microbial inoculation significantly (*p* < 0.05) increased the crude protein, crude polysaccharide, total amino acid, and essential amino acid contents of the fruiting bodies. The fruiting bodies of the first flush of ST treatment possessed the highest umami amino acid content and equivalent umami concentration value. Furthermore, microbial inoculation significantly (*p* < 0.05) enhanced the scavenging ability of crude polysaccharides toward free radicals. The results indicate that microbial inoculation has many benefits for the composting cultivating process of oyster mushrooms and good application prospects.

## 1. Introduction

The oyster mushroom (*Pleurotus* spp.) is one of the most widely cultivated edible fungi in the world, and it is also the third-largest commercially cultivated variety in China [1]. According to the China Edible Fungi Association (CEFA), China’s annual production of oyster mushrooms was 6.11 million tons in 2021. The oyster mushroom is tender, delicious, nutritious, and rich in protein, dietary fiber, vitamins, and mineral elements [2]. Moreover, oyster mushrooms contain various bioactive compounds, such as polysaccharides and terpenoids, which endow them with medicinal activities, including antioxidant, antitumor, anti-inflammatory, antibacterial, hypoglycemic, hyperlipidemia, and hypotensive properties [3,4].

Furthermore, oyster mushroom cultivation plays an important role in promoting farmers’ income growth owing to their broad substrates and simple cultivation techniques [1,5]. At present, there are three main cultivation modes for oyster mushrooms: raw material cultivation, sterilization cultivation, and composting cultivation [6,7]. In the composting cultivation process, the raw materials are first subjected to a short-term composting treatment (5–10 days) followed by conventional cultivation processes for oyster mushrooms [1,8]. In recent years, composting cultivation for oyster mushrooms has been very popular in rural China. It can improve mushroom yield and reduce the contamination rate, equipment investment, and energy consumption [6,9]. Moreover, the yield and quality of mushrooms are also closely related to cultivation substrates, composting time, compost properties, and microbial communities [2,10,11]. Meanwhile, there is a lack of research on the nutritional quality and bioactive compounds of oyster mushrooms cultivated by short-term composting processes.

In recent years, microbial inoculants have been widely applied in composting processes, which can improve composting efficiency by secreting high levels of extracellular enzymes [12]. The inoculation of *Bacillus* can indirectly affect the degradation of lignocellulose in rice straw composting by changing the bacterial community [13]. Moreover, the combined microbial agent VT1000 can increase the relative abundance of thermophilic bacteria and reduce that of pathogens during the composting process with cow manure and wheat straw [14]. Most research on microbial inoculants has mainly focused on the composting process of agricultural and forestry waste, with few reports on further steps in using the compost products, especially for composting cultivation of oyster mushrooms. In our previous study, we obtained an efficient thermophilic lignocellulosic degrading Actinomycetes *Streptomyces thermoviolaceus* BUA-FM01 during the short-term composting process for oyster mushroom cultivation. In this study, we aimed to evaluate the effect of microbial inoculation during the short-term composting process on the yield and nutrients of the obtained oyster mushrooms. *Pleurotus ostreatus* was cultivated using composted substrates with or without microbial inoculation during composting. The yield, biological efficiency (BE), agronomic traits of single fruits, nutritional quality, flavor composition, and antioxidant activity of fruiting bodies in different flushes were further evaluated. This study is conducive to a deeper understanding of the effects of composting cultivation technology on the nutritional quality of oyster mushrooms and promotes further optimization of the technology.

## 2. Materials and Methods

### 2.1. Materials

A thermophilic lignocellulosic degrading strain, *S. thermoviolaceus* BUA-FM01, was isolated during the short-term composting process and preserved in the laboratory of edible and medicinal fungi, Beijing University of Agriculture (BUA). A strain of the oyster mushroom *Pleurotus ostreatus*, “HeiPing 17-1”, was generously donated by Henan Academy of Agricultural Sciences (Henan, China). Millet, corncob, cottonseed hull, wheat bran, and lime were purchased from local markets. Authentic 5′-nucleotides were purchased from Shanghai Anpu Experimental Technology Co., Ltd. (Shanghai, China). Other chemical reagents were purchased from Sino Pharmaceutical Chemical Reagent Co., Ltd. (Shanghai, China).

### 2.2. Preparation of Microbial Inoculation

The *S. thermoviolaceus* BUA-FM01 strain was inoculated into a liquid Luria–Bertani (LB) medium (5%, *v*/*v*), incubated in a constant temperature shaker at 50 °C and 150 rpm for 24 h, and then centrifuged at 5000 rpm for 10 min. The cells were gathered and resuspended with sterile water to approximately 1.0 × 10^8^ CFU/mL.

### 2.3. Composting Process and Mushroom Cultivation

The grain spawn of oyster mushroom was prepared using 98% millet and 2% lime with sterilization at 121 °C for 120 min. A pilot-scale composting experiment was performed with the composting formula with corncob (42.5%), cottonseed hull (42.5%), wheat bran (13.0%), and lime (2.0%). The initial moisture and pH were adjusted to approximately 60–65% and 6.8–7.2, respectively [1]. BUA-FM01 inoculation was added into water with a final dosage of 2.0% (wet weight ratio) and numbered as ST, while the treatment inoculated with the same amount of water was treated as the control (CK). The uniformly stirred raw materials were stacked into trapezoidal piles with approximately 5.0 m length, 0.8 m height, 1.0 m top width, and 1.5 m bottom width. After the piles were completed, holes were drilled in the piles to promote ventilation during the composting process [2,8]. The composting process was carried out for 6 days, and the piles were turned over on days 2, 4, and 6, respectively. During the turning, water in the piles was readjusted to maintain a moisture content (MC) of approximately 60–65%. When the composting was finished, the mature compost was placed in polyethylene plastic bags (22 cm × 48 cm, 3.0 kg wet weight each), sterilized at 100 °C for 1 h, and then naturally cooled to room temperature. Each treatment contained 300 cultivation bags and was further randomized into three equal groups. Subsequently, the cultivation bags were inoculated using grain spawn (2%) and incubated at 22–25 °C in the dark until the mycelia completely colonized the bags. Eventually, the colonized bags were moved into a greenhouse (20–25 °C, humidity of 90–95%) for mushroom harvesting. The contamination rate (CR) during mushroom cultivation was calculated as follows: CR (%) = the number of contaminated bags per 100 bags/100 bags × 100% [1].

### 2.4. Physicochemical Properties of Composted Substrates

Before the mushroom inoculation, the sterilized substrates were collected from the cultivation bags for the determination of physicochemical properties, including pH, electrical conductivity (EC), and MC, total carbon (TC), and total nitrogen (TN) contents [6].

### 2.5. Agronomic Traits and Contamination Rate

The fresh fruiting bodies of each treatment were collected at the first (FF), second (SF), and third (TF) flushes, respectively. Then, the yield and biological efficiency (BE) were calculated. Yield (kg/100 bags) was the total fresh weight of mushrooms harvested from 100 bags of each flush. BE (%) = the fresh weight of mushrooms harvested from 100 bags/the dry weight of the relative substrate × 100%. The contamination rate (CR) is the proportion of contaminated bags to total mushroom bags calculated as CR (%) = number of contaminated bags/100 bags [1]. The agronomic traits of single fruit were determined with the first flush samples, including single weight, cap thickness, cap diameter, color of cap, stipe length, stipe diameter, and MC [15].

### 2.6. Main Nutritional Qualities

The fruiting bodies of each flush were dried at 60 °C with a blast dryer until they reached a constant weight; then, they were pulverized, sieved (40-mesh sieve), and stored at 4 °C for further analysis. The crude protein, crude fiber, crude fat, and crude polysaccharide contents were determined [2]. The crude protein content was determined by automatic Kjeltec nitrogen analyzer (Kjeltec8400, FOSS, Hilleroed, Denmark). The crude fiber content was determined by the acid–base digestion method. The crude fat content was determined by the Soxhlet extraction method. The crude polysaccharide content was determined by the ethanol precipitation method.

### 2.7. Assay of Amino Acid Composition

The amino acid composition of dried fruiting bodies was determined using an automatic amino acid analyzer (L-8800, Hitachi, Tokyo, Japan) [10]. In brief, the mushroom powder (0.03 g) was hydrolyzed at 110 °C for 22 h in a threaded test tube containing 10 mL of 6 mol/L HCl and 5 mg/mL of phenol. Subsequently, the hydrolysate was filtered into a 50 mL volumetric flask and diluted with deionized water. The 2 mL hydrolysate was dried at 40 °C and then redissolved in 2 mL deionized water. After repeating the drying and dissolution processes three times, the dried samples were dissolved with 0.02 mol/L HCl (2 mL) and filtered with a 0.22 μm filter membrane for analysis.

### 2.8. Assay of 5’-Nucleotides

The contents of 5’-nucleotides of different treatments were analyzed using dried mushroom powders by HPLC analysis using a 254 nm diode array detector (Prominence SPD-M20A) and a C18 column (250 mm × 4.6 mm, 5 μm, Agilent, USA) at a flow rate of 1.0 mL/min. The injection volume was 20 μL, and the column temperature was 30 °C. The mobile phase consisted of 50 mmol/L phosphate buffer and methanol (97: 3, *v*/*v*) [16]. Each 5’-nucleotide was identified using the authentic 5’-nucleotide (Shanghai Anpu Experimental Technology Co., Ltd., Shanghai, China).

### 2.9. Equivalent Umami Concentration (EUC) Value

The EUC value is the concentration of monosodium glutamate (MSG), which is equivalent to the umami amino acid and 5’-nucleotide synergistic umami intensity. The EUC value was expressed as g MSG/100 g dry weight and calculated with the following equation [10]:

*Y* = ∑*a_i_b_i_* + 1218(∑*a_i_b_i_*)⋯(∑*a_j_b_j_*)

where *Y* is the EUC value of the samples; *a_i_* is the concentration (g/100 g dry weight) of the respective umami amino acid (UAA; glutamic acid: Glu; or aspartic acid: Asp); *a_j_* is the concentration (g/100 g dry weight) of each umami 5′-nucleotide (5′-adenosine monophosphate: 5′-AMP; 5′-guanosine monophosphate: 5′-GMP; 5′-inosine monophosphate: 5′-IMP; 5′-xanthosine monophosphate: XMP); *b_i_* is the relative umami concentration (RUC) for each UAA to MSG (Glu: 1, Asp: 0.077); *b_j_* is the RUC for umami 5′-nucleotides (5′-AMP, 0.18; 5′-GMP, 2.3; 5′-IMP, 1; 5′-XMP, 0.61); and 1218 is the synergistic constant based on the concentration of g/100 g dry weight used [10,17].

### 2.10. Assay of Antioxidant Activity

The antioxidant activity of each treatment was evaluated using crude polysaccharides of fruiting bodies by the 1,1-diphenyl-2-picrylhydrazyl free radical (DPPH·) and hydroxyl free radical (·OH), respectively. The crude polysaccharide samples were dissolved in distilled water at the final concentrations of 1.0, 2.0, 5.0, and 10.0 mg/mL, respectively. The DPPH· and ·OH scavenging ability were determined by DPPH free radical capacity test kit (BC4750, Solarbio, Beijing, China) and hydroxyl free radical capacity test kit (BC1320, Solarbio, China), respectively [18,19].

### 2.11. Statistical Analysis

All data were presented as mean ± standard deviation (mean ± SD). The significance of the different treatments was tested by one-way ANOVA using SPSS (v.25.0). The significant differences in the average values of the same flush for each treatment were analyzed using Student’s *t*-test. When analyzing the differences in the various flushes of the same treatment, one-way ANOVA was carried out, and the means were compared using Tukey’s test. Statistically significant differences were accepted at the minimum probability level of *p* < 0.05.

## 3. Results and Discussion

### 3.1. Physicochemical Properties of Composted Substrates

Before the mushroom inoculation, the physicochemical properties of the composted substrates were summarized, as shown in Table 1. The TN content and C/N ratio are important indicators for evaluating the quality of cultivation substrates [7,20]. After composting, the TN and C/N ranges of the two treatments were 1.74–1.75% and 27.16–27.49%, respectively, meeting the nutritional requirements for the growth of oyster mushrooms [1,20,21]. Microbial inoculation significantly (*p* < 0.05) reduced the TC and C/N ratio of the composted substrates, while there was no significant (*p* < 0.05) difference in pH, EC, and TN. This indicates that more carbohydrates were consumed in the ST treatment, resulting in a lower C/N ratio. Both treatments represented relatively low EC (much lower than 4.0 mS/cm), which would not be harmful to mushroom mycelial growth [1]. A previous study reported that inoculation with the brown-rot fungus *Gloeophyllum trabeum* during manure–straw composting promoted lignocellulose degradation and increased the maturity degree of composting [22]. The strain *S. thermoviolaceus* BUA-FM01 was isolated from the thermophilic stage of the short-term composting process for oyster mushroom cultivation and possessed highly efficient thermophilic lignocellulosic degrading activity. The TC of ST treatment was significantly lower than CK, suggesting that microbial inoculation during the short-term composting process enhanced the lignocellulose degradation of substrates. Moreover, a moderately lower C/N ratio is more favorable for mycelia growth in oyster mushrooms [7]. The physicochemical properties of the substrates are closely related to the yield and nutritional quality of mushrooms [23]. In this study, microbial inoculation improved the physicochemical properties of substrates and may further improve the yield and quality of oyster mushrooms. In addition, the MC of ST treatment was significantly lower than that of CK treatment. This may be because the ST treatment lost more water during the composting process.

### 3.2. Agronomic Traits and Contamination Rate

The yield, BE, and CR of fruiting bodies grown on the CK and ST treatments are shown in Table 2. Microbial inoculation significantly (*p* < 0.05) increased the yields of the FF and SF, which were 1.10 and 1.06 times higher than that of the CK treatment, respectively. Moreover, the ST treatment demonstrated significantly (*p* < 0.05) higher total yield (113.71 ± 0.75 kg/100 bags) and total BE (110.97%) than those of the CK treatment. A previous study revealed that adding supplementary materials (moringa leaf powder) to the substrate was an efficient way to increase the yield and nutrition of fruiting bodies [24]. *P. ostreatus* supplemented with 15% spent mushroom substrate of *Lentinus edodes* reached the highest BE of 107%, while that of the control treatment was 66% [25]. The addition of 6% moringa leaf powder can also significantly increase the BE of king oyster mushroom *Pleurotus eryngii* (from 45.15% for the control to 69.23%) [24]. In this study, microbial inoculation during the composting process improved the yield of oyster mushrooms, which enriches the type of supplements for mushroom production. In addition, microbial inoculation had no significant effect on the yield and BE of TF. As the harvesting frequency increased, the yield of both treatments significantly (*p* < 0.05) decreased, which may be related to the nutrient consumption of mushroom mycelia in the substrate [10].

It is worth mentioning that microbial inoculation also significantly (*p* < 0.05) reduced the CR during mushroom cultivation (from 3.51% to 2.94%, a decrease of 16.23%). It is precisely because the composting process can effectively reduce the CR of mushroom sticks that the composting cultivation technique of oyster mushrooms has been widely used in China in recent years [6,8]. A combined microbial agent containing *Bacillus*, *Pseudomonas*, and *Oceanobacillus* reshaped the microbial community composition during kitchen waste composting [26]. *Streptomyces griseorubens* JSD-1 inoculant also significantly reshaped the microbial communities during co-composting of swine manure and rice straw [27]. This indicates that the microbial inoculation of *S. thermoviolaceus* BUA-FM01 may reshape microbial communities during the composting process and improve the anti-infection ability of matured substrate toward weed molds.

**Figure 1:**
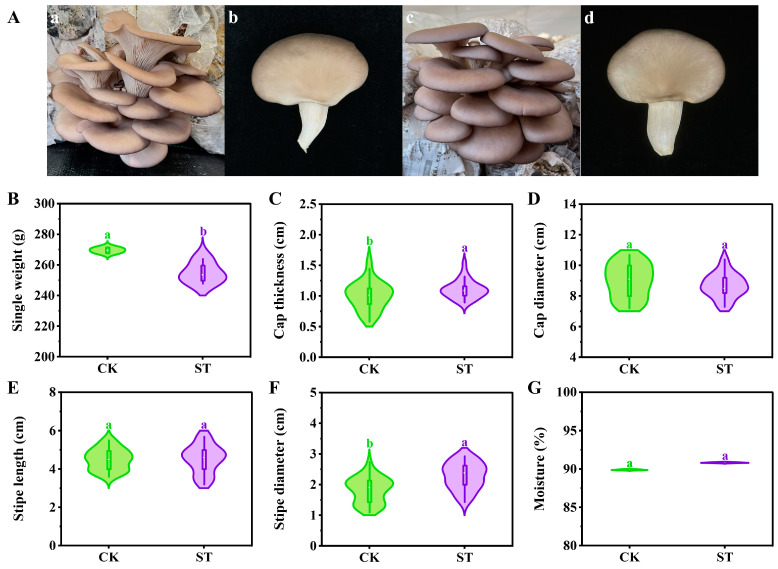
Morphological characteristics and agronomic traits of single fruit of *P. ostreatus* grown on different substrates. CK: control treatment; ST: inoculated treatment; (**A**): a and b for CK; c and d for ST; different lowercase letters in (**B**–**G**) show the discrepancy between treatment groups. All data are expressed as mean ± SD (*n* = 18).

Furthermore, the morphological characteristics and agronomic traits of single fruits in the FF are shown in Figure 1. The cap color is one of the most important commercial characteristics of oyster mushrooms, and dark colors usually have a higher market recognition [28]. The cap color in the ST treatment (Figure 1A(c,d)) was gray-black, while that of the CK treatment (Figure 1A(a,b)) was gray-white. Grey-black oyster mushrooms are much more popular in the market than light-colored ones, indicating that microbial inoculation can increase the market recognition of oyster mushrooms. The pigment of *P. ostreatus* is mainly composed of eumelanin and phaeomelanin in different proportions [28]. Microbial inoculation may affect the synthesis pathway of pigments and the ratio of the two melanins, thus changing the cap color. In terms of single-fruit agronomic traits, the ST treatment showed significantly (*p* < 0.05) lower single weight (259.20 g) and higher cap thickness (1.18 cm), which is also in line with the market preferences for oyster mushrooms.

### 3.3. Main Nutritional Qualities

The main nutritional qualities of different flushes are shown in Table 3. Microbial inoculation significantly (*p* < 0.05) increased the crude protein content of the FF and SF and the crude polysaccharide content of the FF. The crude protein content of the first three flushes was in the range of 22.70–25.22%. The crude protein contents of the FF and SF grown on the ST treatment were 25.22% and 25.16%, respectively, which were 4.76% and 10.17% higher than that of the CK treatment. Mushrooms are rich in proteins, polysaccharides, and dietary fibers [24]. Previous studies have revealed that the protein content of the fruiting bodies of mushrooms is most susceptible to substrate type and nitrogen content [10,29]. The crude protein content of *P. eryngii* varied from 17.56% to 20.41% when cultivated on corn-stalk-based substrates [21]. The oyster mushrooms *Pleurotus columbinus*, *P. ostreatus*, and *Pleurotus sajor-caju* possessed crude protein content of 15.70–23.71%, 20.90–24.36%, and 18.03–29.40%, respectively, when they were grown on different lignocellulosic wastes [30]. The crude protein contents of *Pleurotus cystidiosus* and *P. ostreatus* were 15.68–24.54% and 19.52–29.70%, respectively, when grown on seven substrate formulas with different proportions of sawdust, corncob, and sugarcane bagasse [31]. This indicates that the crude protein content of oyster mushrooms produced by the composting cultivation process is similar to that of non-composting cultivation processes, and microbial inoculation can significantly improve the crude protein content.

Moreover, the crude polysaccharide content of the first three flushes ranged from 5.37% to 10.70%. The crude polysaccharide content of the FF grown on the ST treatment was 10.70%, which was 10.89% higher than that of the CK treatment. Mushroom polysaccharides possess antitumor, immune regulation, antioxidation, anti-inflammatory, antiviral, antidiabetic, and other bioactive properties [4,32]. The crude polysaccharide contents of *P. eryngii* and *Pleurotus floridanus* were 2.05–5.05% and 3.41–5.66%, respectively, when cultivated on different substrates [2,21]. The frequency of collection also affects the nutritional quality of mushrooms. The crude polysaccharide contents of the golden oyster mushroom *Pleurotus citrinopileatus* were 6.70–7.35% and 4.16–6.06%, respectively, when collected for three and four flushes [5]. In this study, the crude polysaccharide contents of the FF of both treatments were significantly higher than the other two flushes.

The dietary fiber in mushrooms can improve intestinal digestive function and reduce blood glucose and cholesterol levels [33,34]. Microbial inoculation had no significant (*p* < 0.05) effects on crude fiber and fat contents. They were in the range of 11.26–18.11% and 1.24–1.39%, respectively, which were close to previous reports [2,5,21,31]. In addition, the fruiting bodies of the FF in both treatments manifested the best nutritional qualities, including the highest crude protein, crude polysaccharide, and crude fiber contents, while there was no significant (*p* < 0.05) difference in the crude fat content among different samples.

### 3.4. Amino Acid Composition

The amino acid composition of different flushes grown on CK and ST treatments is shown in Table 4. Microbial inoculation significantly (*p* < 0.05) improved the contents of total amino acids (TAAs), essential amino acids (EAAs), non-essential amino acids (NEAAs), and most of the assayed amino acids of the FF and SF. The TAA and EAA contents of different flushes grown on the ST treatment were 149.75–258.42 and 62.95–107.50 mg/g, respectively, while those of the CK treatment were 142.35–163.88 and 59.15–67.50 mg/g. The TAA and EAA contents of the FF grown on the ST treatment were 258.42 and 107.50 mg/g, respectively, which were 57.66% and 59.26% higher than that of the CK treatment. *P. floridanus* grown on peach-sawdust-based substrates possessed TAA and EAA ranges of 139.44–158.44 and 58.45–67.18 mg/g [2]. The amino acid content of the fruit bodies is directly related to the physicochemical properties of the culture substrates [7,20]. In this study, microbial inoculation significantly changed the composition of the cultivation substrate (TC and C/N ratio) and, furthermore, significantly increased the amino acid content and composition of the mushrooms.

Amino acids and 5’-nucleotides are considered to be the main contributors to mushroom flavor [10,35]. Moreover, the amino acids can be divided into umami, sweetness, bitterness, and tasteless amino acids, according to their umami characteristics, of which umami and sweet amino acids are considered to be the main flavor components [11,16]. In this study, microbial inoculation significantly (*p* < 0.05) improved the umami amino acid contents of glutamic acid (Glu) and aspartic acid (Asp) of the FF, which were 46.15% and 74.71% higher than that of the CK treatment, respectively. The FF fruiting bodies in the ST treatment demonstrated the highest content of umami and sweetness amino acids (73.36 and 65.66 mg/g, respectively), indicating that oyster mushrooms inoculated with microbial agents had a more delicious taste. In addition, the amino acid contents of both ST and CK treatments significantly (*p* < 0.05) decreased with the number of harvests, which was similar to the results of main nutritional qualities (Table 3).

### 3.5. 5’-Nucleotides and Equivalent Umami Concentration (EUC) Value

The contents of five 5′-nucleotides (including 5’-AMP, 5’-GMP, 5’-IMP, 5’-UMP, and 5’-CMP) of different flushes grown with CK and ST treatments are shown in Table 5. The 5’-AMP was the most abundant nucleotide component in both ST and CK treatments, followed by 5′-UMP. Previous studies have shown that the composition and content of 5′-nucleotides vary among different mushrooms [36,37]. The 5’-AMP contents of the commercial mushrooms *P. cystidiosus*, *P. ostreatus*, and *Flammulina velutipes* were 1.56, 4.37, and 0.42–0.53 mg/g, respectively [36]. Moreover, nucleotides play an important role in enhancing the umami taste of mushrooms; 5’-AMP and 5’-GMP taste more intense, so they are also called umami 5‘-nucleotides [10,37]. 5’-AMP provides sweetness to mushrooms and suppresses bitterness, while 5’-GMP can provide a meaty flavor and has a stronger umami-enhancing effect than MSG [16]. In this study, the 5’-AMP contents of the FF and SF grown on the ST treatment were 3.71 and 3.85 mg/g, respectively, which were 22.52% and 21.87% higher than that of the CK treatment. This indicates that microbial inoculation can significantly (*p* < 0.05) increase the 5’-AMP content and improve the taste of oyster mushrooms. On the other hand, the 5’-UMP content of the FF and SF in the CK treatment was significantly (*p* < 0.05) higher than that in the ST treatment.

The synergistic effect between umami amino acids and 5′-nucleotides plays an important role in the flavor of mushrooms [10,35]. The umami taste of mushrooms can be divided into four levels according to the MSG value: >1000% (>1000 g MSG/100 g dry weight), 100–1000%, 10–100%, and <10% [17,38]. In this study, the EUC values ranged from 175.40 to 394.68 g MSG/100 g dry weight in the ST treatment and from 167.42 to 219.79 g MSG/100 g dry weight in the CK treatment. This indicates that oyster mushrooms cultivated with composting possess a fairly high umami taste. Microbial inoculation significantly (*p* < 0.05) increased the EUC values of the FF and SF in the ST treatment by 79.57% and 37.26%, respectively, compared with the CK treatment. *P. pulmonarius* showed an EUC value range of 72.31–116.73 g MSG/100 g dry weight when grown on three forestry wastes [10]. The EUC value of the wild Croatian form of *P. ostreatus* was 150.55 g MSG/100 g dry weight [39]. This indicates that the oyster mushrooms produced by the composting cultivation process may have a higher umami taste than wild or sterilized cultivated oyster mushrooms.

### 3.6. Evaluation of Antioxidant Activity

**Figure 2:**
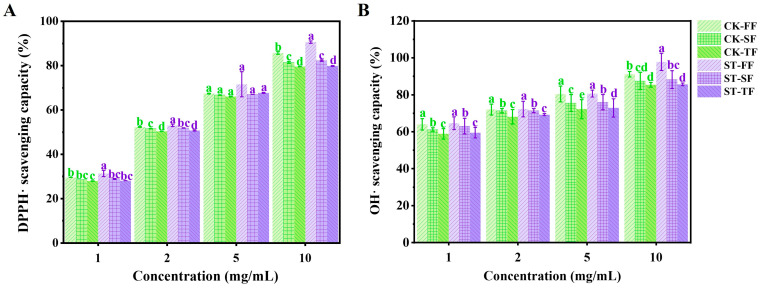
Effect of microbial inoculation on antioxidant capacity of *P. ostreatus* fruiting bodies from different flushes. (**A**) DPPH. (**B**) OH. CK: control treatment; ST: inoculated treatment; FF: first flush; SF: second flush; TF: third flush; different lowercase letters show the discrepancy between treatment groups in the same flush. All data are expressed as mean ± SD (*n* = 6).

Numerous studies have revealed that mushroom polysaccharides present strong antioxidant activity and can act as natural antioxidants to protect cells from oxidative damage [32,40]. The antioxidant activities of crude polysaccharides of *P. ostreatus* grown on CK and ST substrates were analyzed by measuring the DPPH· and ·OH scavenging capacity, respectively; the results are shown in Figure 2. The scavenging ability toward DPPH· and ·OH of crude polysaccharides increased with increasing concentration. The crude polysaccharides of the FF in the ST treatment at a concentration of 10 mg/mL showed the highest scavenging capacity toward DPPH· and ·OH, with values of 90.65% and 97.54%, respectively, which were significantly (*p* < 0.05) higher than that of the CK treatment. However, at doses of 1–5 mg/mL, the ST and CK treatments mainly showed no significant (*p* < 0.05) differences. On the other hand, the antioxidant activity of both treatments decreased significantly (*p* < 0.05) with the increase in harvest times (flushes). This indicates that the antioxidant activity of fruit body polysaccharides decreased with the increase in harvest times. Notably, the scavenging capacity toward ·OH of the six assayed crude polysaccharides at a concentration of 1.0 mg/mL ranged from 57.42 to 65.45%, which was much higher than that of an isolated polysaccharide from *Pleurotus djamor* [41]. The water-soluble crude polysaccharides and the further-purified proteoglycan (8.0 mg/mL) of *P. ostreatus* fruiting bodies showed scavenging abilities toward ⋅OH of 40.5% and 70.6%, respectively [42]. As the concentration increased from 1 to 5 mg/mL, an acidic polysaccharide from the fruiting bodies of *Pleurotus eous* showed a scavenging capacity toward ⋅OH of 30.20–82.55% [43]. Moreover, microbial inoculation significantly (*p* < 0.05) improved the crude polysaccharide yield of the FF (Table 3). This indicates that the oyster mushrooms produced by the composting cultivation process demonstrate considerably high antioxidant activity, and microbial inoculation can enhance this activity of the FF in the high-dose group (10 mg/mL).

## 4. Conclusions

In conclusion, the microbial inoculant *S. thermoviolaceus* BUA-FM01 promoted the degradation of organic matter and reduced the C/N ratio of the substrate during composting. Microbial inoculation significantly improved the total yield, BE, and agronomic traits of single fruits, including cap color and thickness, and significantly inhibited the CR. Moreover, microbial inoculation promoted the nutritional qualities of the oyster mushroom, including crude protein, crude polysaccharide, TAA, and EAA contents. Furthermore, microbial inoculation significantly improved flavor compounds, including umami amino acids and 5’-AMP contents, as well as the scavenging capacity of crude polysaccharides toward DPPH· and ·OH. The results contribute to the nutritional evaluation of oyster mushrooms produced by composting cultivation processes and the development of new additives for composting.

## Figures and Tables

**Table 1:** Physicochemical properties of composted substrate.

Treatment	pH	EC (mS/cm)	TC (%)	TN (%)	C/N Ratio	MC (%)
CK	6.22 ± 0.05 a	1.32 ± 0.04 a	47.99 ± 0.07 a	1.75 ± 0.00 a	27.49 ± 0.06 a	64.24 ± 0.54 a
ST	6.23 ± 0.03 a	1.35 ± 0.01 a	47.38 ± 0.07 b	1.74 ± 0.00 a	27.16 ± 0.04 b	61.96 ± 0.78 b

CK: control treatment; ST: inoculated treatment; EC: electrical conductivity; TC: total carbon content; TN: total nitrogen content; MC: moisture content. Values within columns followed by different letters are significantly different (*p* < 0.05). All data are expressed as mean ± SD (*n* = 6).

**Table 2:** Effects of microbial inoculation on yield, biological efficiency, and contamination rate of *P. ostreatus* fruiting bodies.

Treatment	Flush	Yield (kg/100 Bags)	BE (%)	Total Yield (kg/100 Bags)	Total BE (%)	CR (%)
CK	FF	38.80 ± 1.36 Ab	38.84 ± 1.36 Ab	106.49 ± 1.35 b	106.60 ± 1.35 b	3.51 ± 0.27 a
	SF	34.91 ± 0.50 Bb	34.95 ± 0.50 Ba			
	TF	32.78 ± 0.74 Ca	32.81 ± 0.74 Ca			
ST	FF	42.71 ± 0.96 Aa	41.68 ± 0.94 Aa	113.71 ± 0.75 a	110.97 ± 0.56 a	2.94 ± 0.10 b
	SF	37.09 ± 1.09 Ba	36.19 ± 1.06 Ba			
	TF	33.92 ± 0.45 Ca	33.10 ± 0.44 Ca			

CK: control treatment; ST: inoculated treatment; FF: first flush; SF: second flush; TF: third flush; BE: biological efficiency; CR: contamination rate. Values within columns followed by different letters are significantly different (*p* < 0.05). Different uppercase letters show the discrepancy between different flushes in the same treatment; different lowercase letters show the discrepancy between different treatments in the same flush. All data are expressed as mean ± SD (*n* = 3).

**Table 3:** Effects of microbial inoculation on main nutritional qualities of *P. ostreatus* fruiting bodies from different flushes.

Treatment	Flush	Crude Protein (%)	Crude Polysaccharide (%)	Crude Fiber (%)	Crude Fat (%)
CK	FF	24.07 ± 0.21 Ab	9.65 ± 0.60 Ab	18.11 ± 0.29 Aa	1.39 ± 0.07 Aa
	SF	22.84 ± 0.21 Bb	5.86 ± 0.55 Ba	14.85 ± 1.04 Ba	1.32 ± 0.08 Aa
	TF	22.70 ± 0.75 Ba	5.47 ± 0.59 Ba	11.26 ± 0.50 Ca	1.29 ± 0.06 Aa
ST	FF	25.22 ± 0.23 Aa	10.70 ± 0.43 Aa	17.90 ± 1.88 Aa	1.36 ± 0.08 Aa
	SF	25.16 ± 0.23 Aa	5.70 ± 0.34 Ba	13.31 ± 1.00 Ba	1.32 ± 0.06 Aa
	TF	22.87 ± 0.22 Ba	5.37 ± 0.47 Ba	11.81 ± 0.50 Ba	1.24 ± 0.09 Aa

CK: control treatment; ST: inoculated treatment; FF: first flush; SF: second flush; TF: third flush. Values within columns followed by different letters are significantly different (*p* < 0.05). Different uppercase letters show the discrepancy between flushes; different lowercase letters show the discrepancy between treatment groups in the same flush. All data are expressed as mean ± SD (*n* = 4).

**Table 4:** Effects of microbial inoculation on amino acid content of *P. ostreatus* fruiting bodies from different flushes.

Amino Acid	Content (mg/g Dry Weight)					
	CK			ST		
	FF	SF	TF	FF	SF	TF
^a,f^ Glu	29.91 ± 3.56 Ab	27.07 ± 0.49 ABb	25.24 ± 0.82 Ba	43.67 ± 0.72 Aa	29.97 ± 0.10 Ba	25.02 ± 0.79 Ca
^c,e^ Val	19.51± 5.88 Ab	19.46 ± 2.40 Aa	15.27 ± 0.50 Ab	29.91 ± 0.37 Aa	18.55 ± 0.06 Ba	17.92 ± 0.54 Ca
^a,f^ Asp	17.04 ± 1.98 Ab	17.78 ± 0.32 Ab	13.94 ± 0.46 Bb	29.68 ± 0.42 Aa	20.45 ± 0.08 Ba	17.46 ± 0.56 Ca
^b,f^ Ser	10.87 ± 1.26 Ab	10.74 ± 0.22 Ab	9.89 ± 0.33 Aa	15.17 ± 0.06 Aa	12.35 ± 0.07 Ba	7.87 ± 0.25 Cb
^c,e^ Leu	10.64 ± 1.35 Ab	10.41 ± 0.12 Ab	10.20 ± 0.35 Aa	18.51 ± 0.29 Aa	12.43 ± 0.05 Ba	10.51 ± 0.33 Ca
^c,f^ Arg	10.04 ± 1.22 Ab	9.98 ± 0.21 Ab	8.48 ± 0.30 Bb	16.58 ± 0.28 Aa	11.34 ± 0.03 Ba	9.77 ± 0.31 Ca
^b,f^ Ala	9.60 ± 1.17 Ab	9.30 ± 0.17 Ab	9.01 ± 0.30 Aa	15.79 ± 0.17 Aa	10.73 ± 0.05 Ba	9.45 ± 0.29 Ca
^d,e^ Lys	9.09 ± 1.12 Ab	8.95 ± 0.17 Ab	8.20 ± 0.28 Ab	15.56 ± 0.23 Aa	10.46 ± 0.03 Ba	9.21 ± 0.29 Ca
^b,f^ Gly	7.44 ± 0.90 Ab	7.25 ± 0.13 Ab	7.27 ± 0.24 Aa	12.28 ± 0.10 Aa	8.22 ± 0.04 Ba	7.18 ± 0.21 Ca
^b,e^ Thr	7.19 ± 0.82 Ab	6.99 ± 0.13 Ab	6.42 ± 0.20 Aa	10.83 ± 0.15 Aa	7.98 ± 0.03 Ba	6.21 ± 0.19 Ca
^c,e^ Phe	6.88 ± 0.84 Ab	6.66 ± 0.13 Ab	6.25 ± 0.20 Ab	11.47 ± 0.15 Aa	7.87 ± 0.02 Ba	6.79 ± 0.21 Ca
^b,f^ Pro	6.41 ± 0.87 Ab	6.48 ± 0.12 Ab	6.09 ± 0.21 Ab	11.60 ± 0.24 Aa	7.62 ± 0.03 Ba	6.41 ± 0.23 Ca
^c,e^ Ile	6.16 ± 0.76 Ab	5.97 ± 0.08 Ab	5.82 ± 0.20 Ab	11.37 ± 0.18 Aa	6.97 ± 0.03 Ba	6.55 ± 0.20 Ca
^d,f^ Tyr	4.51 ± 0.55 Ab	4.09 ± 0.11 Ab	2.83 ± 0.12 Bb	5.57 ± 0.08 Aa	5.01 ± 0.02 Ba	3.27 ± 0.11 Ca
^c,e^ Met	4.27 ± 0.80 Aa	3.61 ± 0.86 Ab	4.18 ± 0.17 Aa	3.41 ± 0.08 Bb	7.19 ± 0.07 Aa	1.89 ± 0.06 Cb
^c,e^ His	3.77 ± 0.46 Ab	3.43 ± 0.08 Ab	2.82 ± 0.09 Bb	6.44 ± 0.06 Aa	4.27 ± 0.04 Ba	3.85 ± 0.14 Ca
^f^ Cys	0.56 ± 0.05 Aa	0.57 ± 0.01 ABb	0.45 ± 0.01 Ba	0.58 ± 0.01 Ba	0.66 ± 0.01 Aa	0.39 ± 0.01 Ca
Umami	46.95 ± 5.54 Ab	44.86 ± 0.81 Ab	39.18 ± 1.28 Bb	73.36 ± 1.15 Aa	50.42 ± 0.17 Ba	42.48 ± 1.35 Ca
Sweetness	41.51 ± 5.01 Ab	40.77 ± 0.76 Ab	38.68 ± 1.27 Aa	65.66 ± 0.64 Aa	46.90 ± 0.22 Ba	37.11 ± 1.17 Ca
Bitterness	61.26 ± 9.79 Ab	59.52 ± 1.89 Ab	53.01 ± 1.80 Ab	97.69 ± 1.41 Aa	68.62 ± 0.22 Ba	57.30 ± 1.78 Ca
Tasteless	13.61 ± 1.68 Ab	13.05 ± 0.28 Ab	11.04 ± 0.47 Bb	21.14 ± 0.30 Aa	15.47 ± 0.06 Ba	12.48 ± 0.40 Ca
EAA	67.50 ± 10.51 Ab	65.48 ± 1.96 Ab	59.15 ± 1.97 Ab	107.50 ± 1.50 Aa	75.72 ± 0.25 Ba	62.95 ± 1.95 Ca
NEAA	96.38 ± 11.56 Ab	93.28 ± 1.77 ABb	83.20 ± 2.77 Ba	150.92 ± 1.99 Aa	106.35 ± 0.41 Ba	86.81 ± 2.76 Ca
TAA	163.88 ± 22.05 Ab	158.76 ± 3.39 Ab	142.35 ± 4.74 Aa	258.42 ± 3.50 Aa	182.08 ± 0.66 Ba	149.75 ± 4.71 Ca

CK: control treatment; ST: inoculated treatment; FF: first flush; SF: second flush; TF: third flush; Umami: umami amino acids; Sweetness: sweetness amino acids; Bitterness: bitterness amino acids; Tasteless: tasteless amino acids; EAA: essential amino acid; NEAA: non-essential amino acid; TAA: total amino acid; ^a^: Umami; ^b^: Sweetness; ^c^: Bitterness; ^d^: Tasteless; ^e^: EAA; ^f^: NEAA. Values within columns followed by different letters are significantly different (*p* < 0.05). Different uppercase letters show the discrepancy between flushes; different lowercase letters show the discrepancy between treatment groups in the same flush. All data are expressed as mean ± SD (*n* = 4).

**Table 5:** Effects of microbial inoculation on 5′-nucleotide contents and EUC values of *P. ostreatus* fruiting bodies from different flushes.

5′-Nucleotides	Content (mg/g Dry Weight)					
	CK			ST		
	FF	SF	TF	FF	SF	TF
5′-AMP	3.028 ± 0.085 Ab	3.159 ± 0.097 Ab	2.856 ± 0.012 Ba	3.710 ± 0.155 Aa	3.850 ± 0.071 Aa	2.986 ± 0.064 Ba
5′-GMP	0.014 ± 0.001 Ba	0.018 ± 0.001 Ab	0.003 ± 0.000 Ca	0.015 ± 0.001 Ba	0.027 ± 0.001 Aa	0.003 ± 0.000 Ca
5′-IMP	0.003 ± 0.000 Aa	0.002 ± 0.000 Cb	0.002 ± 0.000 Ba	0.003 ± 0.000 Aa	0.002 ± 0.000 Ba	0.002 ± 0.000 Ba
5′-UMP	0.526 ± 0.18 Aa	0.308 ± 0.003 Ba	0.296 ± 0.015 Ba	0.255 ± 0.001 Ab	0.209 ± 0.009 Bb	0.144 ± 0.016 Cb
5′-CMP	nd	nd	nd	nd	nd	nd
5′-XMP	nd	nd	nd	nd	nd	nd
Total	3.570 ± 0.079 Ab	3.486 ± 0.098 Ab	3.157 ± 0.026 Bb	3.983 ± 0.156 Aa	4.087 ± 0.081 Aa	3.135 ± 0.053 Bb
EUC (g MSG/100 g)	219.79 ± 17.91 Ab	211.90 ± 7.61 Ab	167.42 ± 4.78 Ba	394.68 ± 11.71 Aa	290.85 ± 4.67 Ba	175.40 ± 6.48 Ca

CK: control treatment; ST: inoculated treatment; FF: first flush; SF: second flush; TF: third flush; EUC: equivalent umami concentration; nd: not detected. Values within columns followed by different letters are significantly different (*p* < 0.05). Different uppercase letters show the discrepancy between flushes; different lowercase letters show the discrepancy between treatment groups in the same flush. All data are expressed as mean ± SD (*n* = 4).

## Data Availability

All experimental data in this study will be made available upon reasonable request from readers.
